# Bibliometric analysis of T‐cells immunity in pulmonary hypertension from 1992 to 2022

**DOI:** 10.1002/iid3.1280

**Published:** 2024-07-05

**Authors:** Xian Chen, Zhe Yan, Qing Pan, Chunxia Zhang, Yakun Chen, Xuzhi Liang, Shaomei Li, Lei Wang

**Affiliations:** ^1^ Department of Nephrology Second Hospital of Hebei Medical University Shijiazhuang China; ^2^ Department of Pulmonary and Critical Care Medicine Second Affiliated Hospital of Xi'an Jiaotong University Xi'an China

**Keywords:** bibliometric analysis, CiteSpace, pulmonary hypertension, T‐cell, Web of Science

## Abstract

**Background:**

Adaptive immunity is an important disease mediator of pulmonary vascular remodeling during pulmonary hypertension (PH) development, especially T‐cells lymphocytes. However, data for bibliometric analysis of T cell immunity in PH is currently vacant. This aimed to provide a comprehensive and visualized view of T‐cells research in PH pathogenesis and to lay a solid foundation for further studies.

**Methods:**

The data was acquired from the Web of Science Core Collection database. Web of Science analytic tool was used to analysis the publication years, authors, journals, countries, and organizations. CiteSpace 6.2.R3, VOSviewer 1.6.16, and Scimago Graphica 1.0.35.0 were applied to conduct a visualization bibliometric analysis about authors, countries, institutions, journals, references, and keywords.

**Results:**

Nine hundred and eight publications from 1992 to 2022 were included in the analysis. The results showed that Humbert Marc was the most prolific author. *American Journal of Physiology Lung Cellular and Molecular Physiology* had the most related articles. The institution with the most articles was Udice French Research University. The United States was far ahead in the article output. Keywords analysis showed that “Pulmonary hypertension” was the most usually appeared keyword in the relevant literature, and included “T‐cells”, “Regulatory T cells”, and “Activated T cell.” “miRNA” of reference co‐citation clustering analysis demonstrated the possible T‐cell immunity activation mechanisms in PH. The most cited literature was published in the European Heart Journal by Galie N in 2016. The strongest citation burst of keyword is “gene expression” and terms such as “vascular remodeling,” “growth,” “proliferation,” and “fibrosis” are among the list, indicating that T‐cells interact with stromal vascular cells to induce pulmonary vascular remodeling. The strongest burst of cited reference is “Galie N, 2016.”

**Conclusions:**

T‐cell immunity is an important pathogenesis mechanism for PH development, which may have interaction with miRNAs and stromal vascular cells, but the possible T‐cell immunity activation mechanisms in PH need to be investigated further.

## Graphical abstract

This work provides readers with a scientific and comprehensive view of the structure of knowledge in the field of T‐cell immunity in pulmonary hypertension, providing research hotspots, frontiers, development trends, and a theoretical basis for subsequent scientific exploration.

## 1 BACKGROUND

Pulmonary hypertension (PH) is characterized by elevated pulmonary artery pressure, which eventually leads to right heart failure and death. PH includes World Health Organization (WHO) Group 1 pulmonary arterial hypertension (PAH), Group 2 PH due to left‐heart disease (PH‐LHD), Group 3 PH related with lung diseases and/or hypoxia (HPH), Group 4 Chronic thromboembolic pulmonary hypertension (CTEPH), and Group 5 PH due to unclear and/or multifactorial mechanisms.^1^ Present estimates suggest a PH prevalence of about 1% of the global population, which increases up to 10% in individuals aged more than 65 years.^2^ PH places a considerable socioeconomic burden on patients, their families, the healthcare system, and society as a whole.^3^ There is currently no cure for PH because of its unclear pathogenesis, although research on a cure is ongoing. Therefore, it is urgently essential to keep up with the current hotspots to explore the pathogenesis of PH.

Up to now, new assessment tools are increasingly available for PH.^4^, ^5^ While due to its complex pathogenesis, although there are some effective treatments,^6^ there is still a lack of very effective treatments for PH, so it is urgent to explore the pathogenesis of PH. Pulmonary vascular remodeling is the key structural alteration in PH and involves changes in the intima, media, and adventitia, often with the interplay of inflammatory/immune cells.^7^, ^8^ Nowadays researchers proved that inflammatory/immune mechanisms are crucial element that can lead to pulmonary vascular remodeling in PH.^9^, ^10^ T lymphocytes is now increasingly recognized to be very important for PH development, and the T‐lymphocyte subsets, which include helper T‐cells (Th cells), cytotoxic T‐cells, and regulatory T‐cells (Tregs), play different roles in PH, especially in the pulmonary vascular remodeling process.^11^, ^12^, ^13^ Due to its importance for the progression of PH, an increasing number of studies have focused on the role of T lymphocytes in PH pathogenesis which remains unclear yet, and efforts have been made to explore novel therapies targeting T lymphocytes to alleviate PH. Therefore, a systematic review and summary of the topical and major findings of T lymphocytes in PH based on the current literature is urgent and necessary at this stage, but it is hard to quickly summarize the underlying role of T lymphocytes in PH process.

Bibliometric analysis is a scientific computer‐assisted review methodology that can identify core research works or authors, as well as their relationship, which could cover all the publications related to a given topic or field.^14^ Bibliometric analysis was first proposed to examine intellectual flow and most influential publications based on the author or citation information. In recent years, bibliometric analysis developed to adopt network analysis and sociometric analysis based on the titles, keywords, and abstract data to identify the research hotspots and frontiers in a specific field, and grasp the future trends in a specific field, which making it play a more and more important role in the field of medicine lately now.^15^, ^16^ However, there is still a lack of data on the bibliometric analysis of T lymphocytes in PH.

In this study, we retrieved T lymphocytes‐related articles in PH field from the Web of Science database and analyzed the literature characteristics and research hotspots using bibliometric analysis tools. The purpose of this study is to provide a comprehensive and visualized view of T lymphocytes research in PH pathogenesis and to lay a solid foundation for further studies.

## 2 METHODS

### 2.1 Data source and establishment

The literature data in this article was collected from the Web of Science Core Collection (WoSCC) on June 26, 2023. The range of search time was from January 1, 1992 to December 31, 2022. In the WoSCC database, the subject term search was used, and we didn't restrain the subject area. The search strategy was: TS = “pulmonary hypertension” AND TS = “T cell”. Only articles and reviews were gathered in our analysis. All electronic searcher were completed on June 26, 2023. XC and LW retrieved and download the data independently, exported the related records and cited references to a plain text file, and stored the data in download_txt format. In case of disagreement between the above two reviewers, a third reviewer is permitted to involved in the discussion. The records were imported into the CiteSpace 6.2.R3, VOSviewer 1.6.16, and Scimago Graphica 1.0.35.0 for visualized analysis. The study was bibliometric research and did not contain any clinical trials and patient informed consent. So, ethics committee approval was not necessary.

### 2.2 Data processing

Microsoft Excel 2019 was employed to complete the plotting and histogram diagram. WoS analytic tool was used to analysis the basic information, such as publication years, authors, journals, countries and organizations. CiteSpace 6.2.R3 was applied to conduct a visualization bibliometric analysis of T‐cells in PH, including co‐occurrence visualization maps of authors, countries, institutions, journals, references, keywords, and performed cluster analysis, emergence analysis of references and keywords, the bursts of keywords and references, and the timeline of keyword clusters and reference clusters. VOSviewer 1.6.16 and Scimago Graphica 1.0.35.0 were used for country co‐authorships analysis and visualization.

Parameters setting for CiteSpace 6.2.R3 were as follows: ①Time slicing:1992–2022; time zone selection (year per silce): 1 year. ②node type: author, country, institution, reference, keywords. ③Selection criteria (g‐index): k = 15%. We choose 15% to prevent the co‐citation network from being too complex. ④Pruning: pathfinder and pruning sliced network were selected, which could precisely extract the key structure of the network by removing the edges that violate the triangle inquality. Cluster analysis was used to reveal the correlation. CiteSpace clustering algorithm, containing LSI algorithm, LLR algorithm, and USR algorithm, was applied to explore research hotspots, find mutation words in the map and grasp the research direction. In our study, we choose the LLR algorithm.

## 3 RESULTS

### 3.1 The trend of annual publication quantity

**Figure 1:**
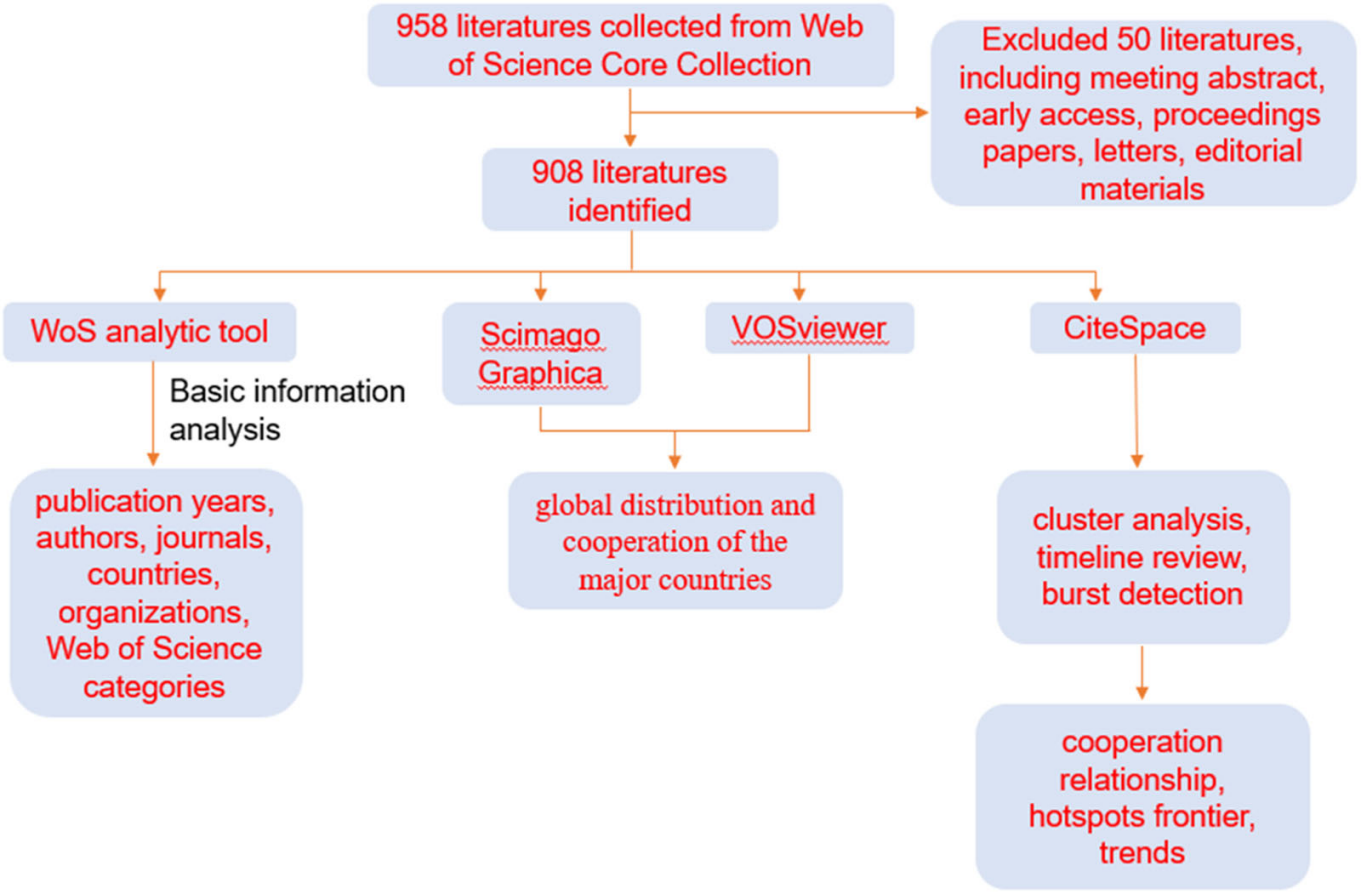
The workflow of bibliometric analysis of T‐cell in pulmonary hypertension.

**Figure 2:**
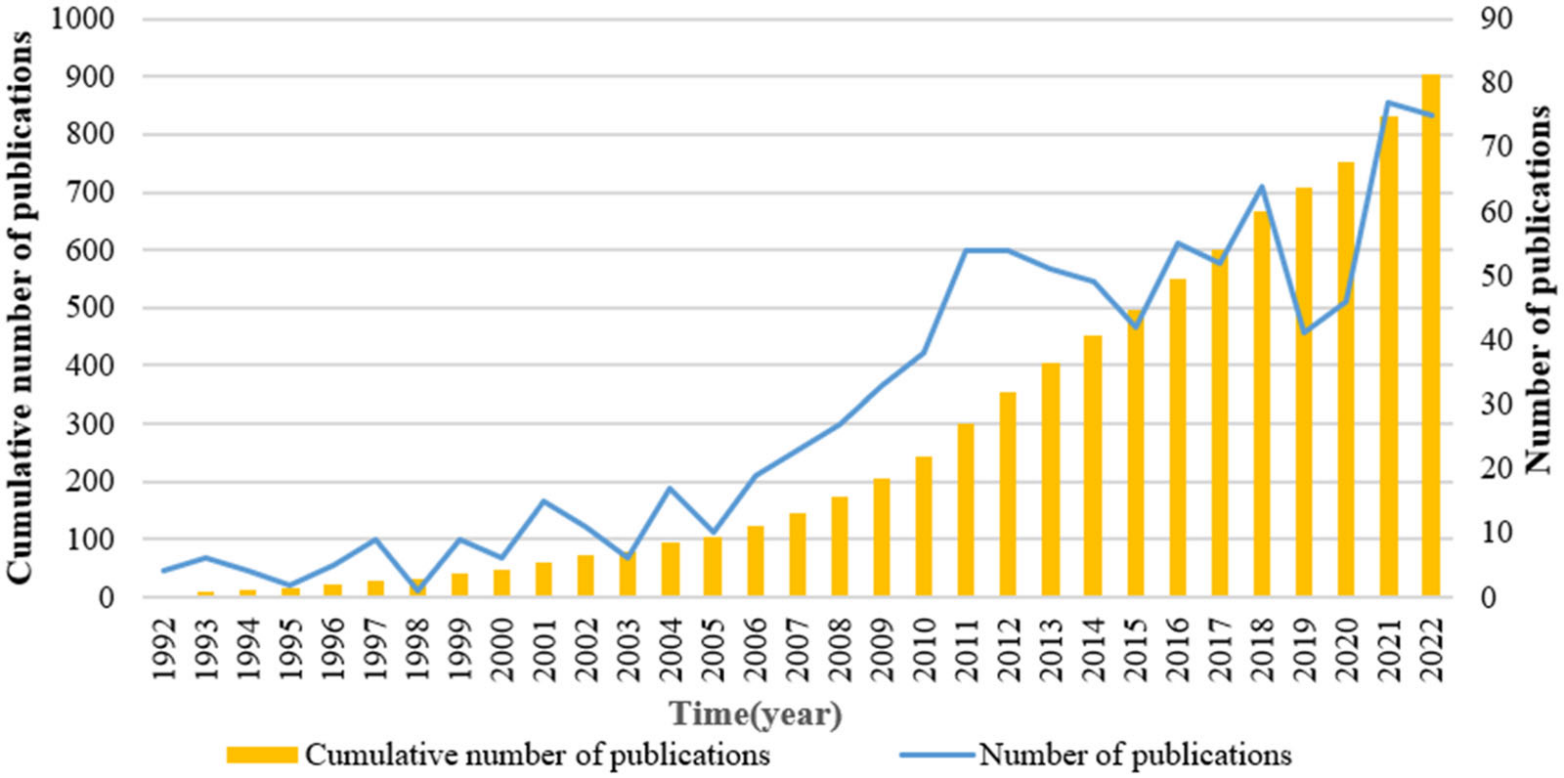
The quantities of annual publications of T‐cell in pulmonary hypertension.

Using the search strategy outline above (Figure 1), a total of 908 publications from 1992 to 2022 were included in the analysis. The annual number of T‐cell and PH‐related publications fluctuated over time. Increasing numbers of researchers started to investigate in this field in 2006, which caused a faster increase in the number of publications, and the cumulative number of publications raised gradually (Figure 2). Although studies on T‐cell and PH have elevated significantly during the recent years, it remains an area that worth exploring further.

### 3.2 Web of Science categories

**Figure 3:**
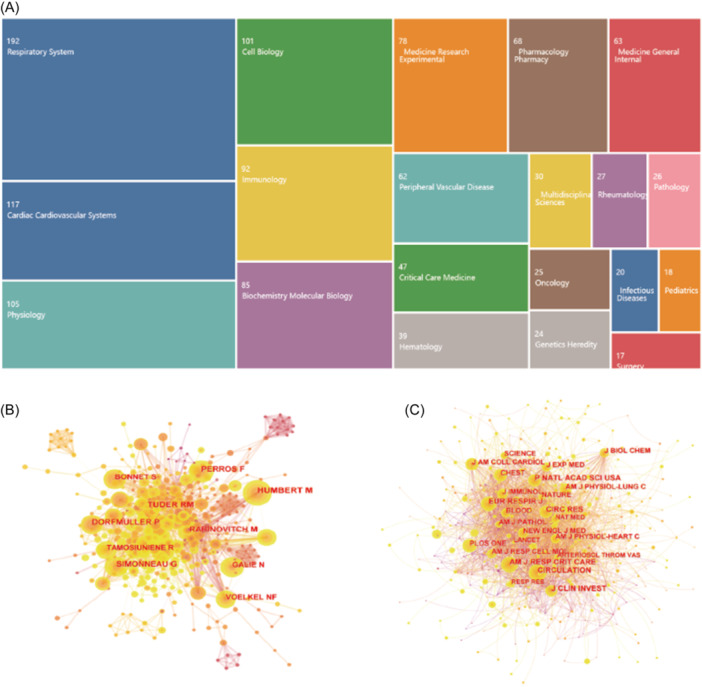
The analysis of categories, cited authors and cited journals of T‐cell in pulmonary hypertension. (A) TOP 20 categories of publications about T‐cell in pulmonary hypertension. (B) The analysis of cited authors of T‐cell in pulmonary hypertension. (C) The analysis of cited journals of T‐cell in pulmonary hypertension.

All the analyzed records were divided into 65 entries of the Web of Science categories. Respiratory System was the largest part, accounting for 21.1% of the records, followed by Cardiac Cardiovascular Systems accounting for 12.8%. TOP 20 categories were shown in Figure 3A.

### 3.3 Analysis of authors and cited authors distribution

Two hundred authors contributed to the 908 literatures about T cell and PH published in WoSCC. Among the top 20 prolific authors, France took the most proportion of 30%, followed by United States with a proportion of 25%. Among the 908 publications, 391 came from United States. Humbert Marc takes the first place with 27 articles, Voelkel Norbert F. followed by with 23 articles, and Perros Frédéric with 20 articles (Table 1). Talking of the cited author, Humbert Marc takes the first place with 197 citations, followed by Tuder RM with 141 citations and Perros Frédéric with 132 citations (Figure 3B).

**Table 1:** The top 20 prolific authors.

Author	Article counts	Organization	Country
Humbert Marc	27	UDICE‐French Research Universities	France
Voelkel Norbert F.	23	University of amsterdam	Netherlands
Perros Frédéric	20	Institut National de la Sante et de la Recherche Medicale	France
Nicolls. Mark	16	Stanford University	USA
Yuan Jason X‐J	14	University of California System	USA
Schermuly Ralph Theo	13	Justus Liebig University Giessen	Germany
Dorfmuller Peter	12	University Hospital of Giessen & Marburg	Germany
Guignabert Christophe	12	Universite Paris Saclay	France
Bosc Laura V Gonzalez	12	University of New Mexico's Health Sciences Center	USA
David Montani	12	Universite Paris Saclay	France
Resta Thomas C.	11	University of New Mexico	USA
Rabinovitch Marlene	11	Stanford University	USA
Bonnet Sebastien	11	Quebec Heart & Lung Institute	Canada
Cohen‐Kaminsky Sylvia	10	Institut National de la Sante et de la Recherche Medicale	France
Seeger Werner	9	Justus Liebig University Giessen	Germany
Kwapiszewska G.	9	Ludwig Boltzmann Institute	Austria
Tu Ly	9	Univ Paris Saclay	France
Weissmann Norbert	8	Univ Giessen & Marburg Lung Ctr UGMLC	Germany
Lopes Antonio Augusto	8	Universidade de Sao Paulo	Brazil
Klepetko, Walter	8	Medical University of Vienna	Austria
Bogaard, Harm Jan	8	Vrije Universiteit Amsterdam	Netherlands

### 3.4 Analysis of journals and cited journals distribution

The articles related with T‐cell and PH are distributed among 200 journals. *American Journal of Physiology Lung Cellular and Molecular Physiology* ranks the first place with 44 publications in the area of T‐cell and PH. It is followed by *American Journal of Respiratory and Critical Care Medical* (27 publications), *American Journal of Respiratory and Molecular Biology* (20 publications), *Frontiers in Immunology* (20 publications), *PLOS* One (16 publications), *American Journal of Physiology Heart and Circulatory Physiology* (14 publications), *European Respiratory Journal* (14 publications), *International Journal of Molecular Sciences* (11 publications), *Pulmonary Circulation* (10 publications), and *Chest* (9 publications). Among the top 16 journals, *Circulation* has the highest impact factor (IF) of 37.8, followed by *American Journal of Respiratory and Critical Care Medical* with the IF of 24.7. *American Journal of Physiology Lung Cellular and Molecular Physiology* with the highest number of publications in the field has an IF of 4.9 Therefore, the vast majority of publications related with T‐cell and PH are of high quality and worth further investigation (Table 2). As for the cited journals, Circulation takes the first place with 529 citations, followed by *American Journal of Respiratory and Critical Care Medical* with 516 citations, the *Journal of Clinical Investigation* with 473 citations and *Circulation Research* with 462 citations (Figure 3C).

**Table 2:** The top 16 most productive journals.

Journal	Publications	IF2022	Publisher	Country
*American Journal of Physiology Lung Cellular and Molecular Physiology*	44	4.9	Amer Physiological SOC	USA
*American Journal of Respiratory and Critical Care Medical*	27	24.7	Amer Thoracic SOC	USA
*American Journal of Respiratory and Molecular Biology*	20	6.4	Amer Thoracic SOC	USA
*Frontiers in Immunology*	20	7.3	Frontiers Media SA	Switzerland
*PLOS One*	16	3.7	Public Library Science	USA
*American Journal of Physiology Heart and Circulatory Physiology*	14	4.8	Amer Physiological SOC	USA
*European Respiratory Journal*	14	24.3	European Respiratory SOC Journals LTD	England
*International Journal of Molecular Sciences*	11	5.6	MDPI	Switzerland
*Pulmonary Circulation*	10	2.6	WILEY	USA
*Circulation*	10	37.8	LIPPINCOTT WILLIAMS & WILKINS	USA
*Chest*	9	9.6	ELSEVIER	Netherlands
*Pulmonary Pharmacology Therapeutics*	9	3.2	Academic Press LTD‐ELSEVIER Science LTD	England
*American Journal of Pathology*	8	6	ELSEVIER SCIENCE INC	USA
*Circulation Research*	7	20.1	LIPPINCOTT WILLIAMS & WILKINS	USA
*Hypertension*	7	8.3	LIPPINCOTT WILLIAMS & WILKINS	USA
*Respiratory Research*	7	5.8	BMC	England

### 3.5 Coauthorship analysis of countries/regions distribution

**Figure 4:**
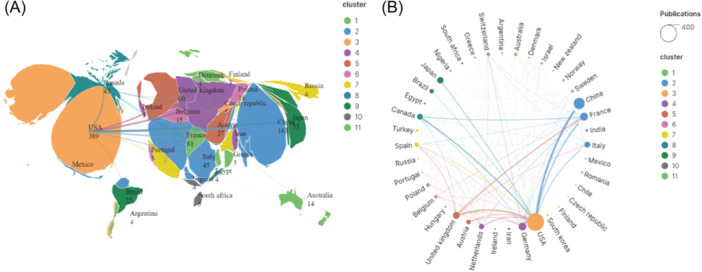
The global distribution and cooperation network of T‐cell in pulmonary hypertension. (A) The global distribution of T‐cell in pulmonary hypertension. (B) The cooperation network of T‐cell in pulmonary hypertension in different countries. The size of the circle represents the number of total publications in different countries, the width of the lines between different countries represents the strength of their cooperation.

Sixty‐eight countries involved in the publication of literatures about T‐cell and PH according to the retrieval results of the WoSCC database. The global distribution and cooperation of the major countries were shown in Figure 4. United States contributes the most with 391 publications, accounting for 43.06% of all the publications, followed by China (158 publications), France (81 publications), Germany (77 publications), England (58 publications), and Japan (54 publications), which is accordance with the author distribution.

### 3.6 Analysis of organization distribution

According to the WoSCC database, almost 200 organizations involved in the research of T‐cell and PH. Udice French Research University took the first place with 66 literatures, followed by Institut National De La Sante Et De La Recherche Medicale Inserm with 55 publications and Assistance Publique Hospitaux Paris with 49 literature. Organizations from France predominate in the field of T‐cell and PH.

### 3.7 Cluster analysis of co‐appearance keyword

**Figure 5:**
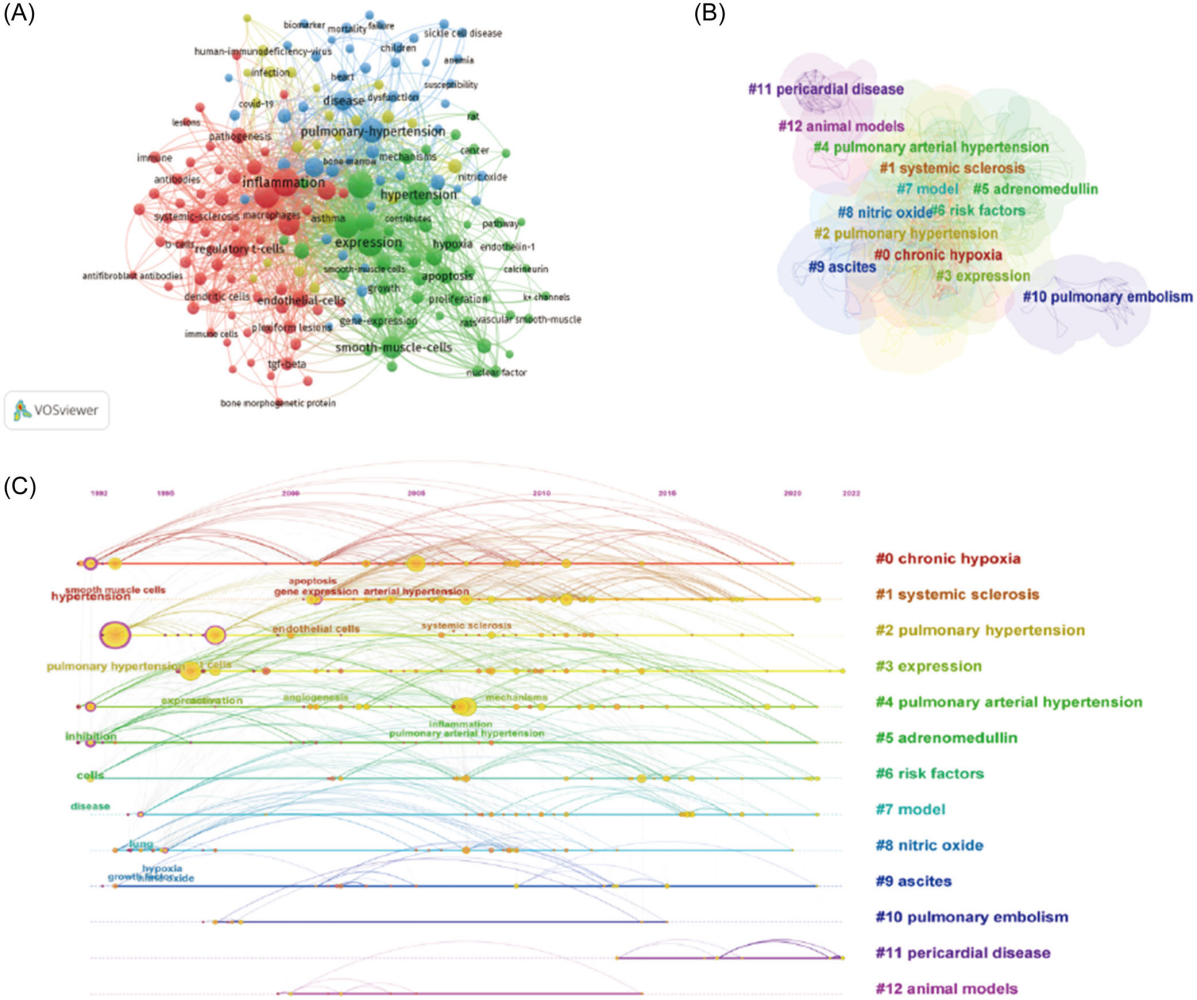
Analysis of keyword co‐appearance of T‐cell in pulmonary hypertension. (A) The co‐appearance of keyword about T‐cell in pulmonary hypertension. (B) The cluster analysis of keyword about T‐cell in pulmonary hypertension. (C) The timeline view of keyword co‐appearance of T‐cell in pulmonary hypertension.

Keywords are a series of words that define what your content is about, and they are the linchpin between what people are searching for and the content you are providing to fill that need. Hence, we can get a general perception of the characteristics and themes of literatures through keyword analysis. The co‐appearance of keyword was shown in Figure 5A and the top 20 most frequent keywords were shown in Table 3. “Pulmonary hypertension” was the most usually appeared keyword in the relevant literature, followed by “pulmonary arterial hypertension,” “expression,” “t cells,” “arterial hypertension,” “smooth muscle cells,” “inflammation,” “hypertension.” Cluster analysis of co‐appearance keyword was conducted (Figure 5B). Clusters located in the top five are chronic hypoxia, systemic sclerosis, PH, expression, and pulmonary arterial hypertension. The keyword co‐occurrence timeline view showed the development and change of keywords in each cluster (Figure 5C).

**Table 3:** Top 20 keywords of pulmonary hypertension and T‐cell.

Ranking	Counts	Centrality	Keywords
1	254	0.14	Pulmonary hypertension
2	164	0.03	Pulmonary arterial hypertension
3	162	0.11	Expression
4	145	0.18	T‐cells
5	122	0.08	Arterial hypertension
6	106	0.11	Smooth muscle cell
7	85	0.05	Inflammation
8	81	0.31	Hypertension
9	67	0.13	Activation
10	65	0.37	Cells
11	61	0.05	Disease
12	59	0.08	Systemic sclerosis
13	56	0.02	Regulatory t cells
14	55	0.12	Endothelial cells
15	47	0.09	Inhibition
16	44	0.11	Lung
17	39	0.17	Nitric oxide
18	36	0.03	Receptor
19	33	0.1	Gene expression
20	30	0.05	Activated t cell

### 3.8 Cluster analysis of co‐cited reference

Reference co‐citation clustering analysis allows us to visually understand relevant research topics and hotspots. Each cluster represents a research frontier in some way, which can prompt relevant scholars to follow the research hotspots. In this circumstance, clustering analysis and timeline view of reference co‐citation were completed by CiteSpace to analyze the research trend of T‐cells in PH.

**Figure 6:**
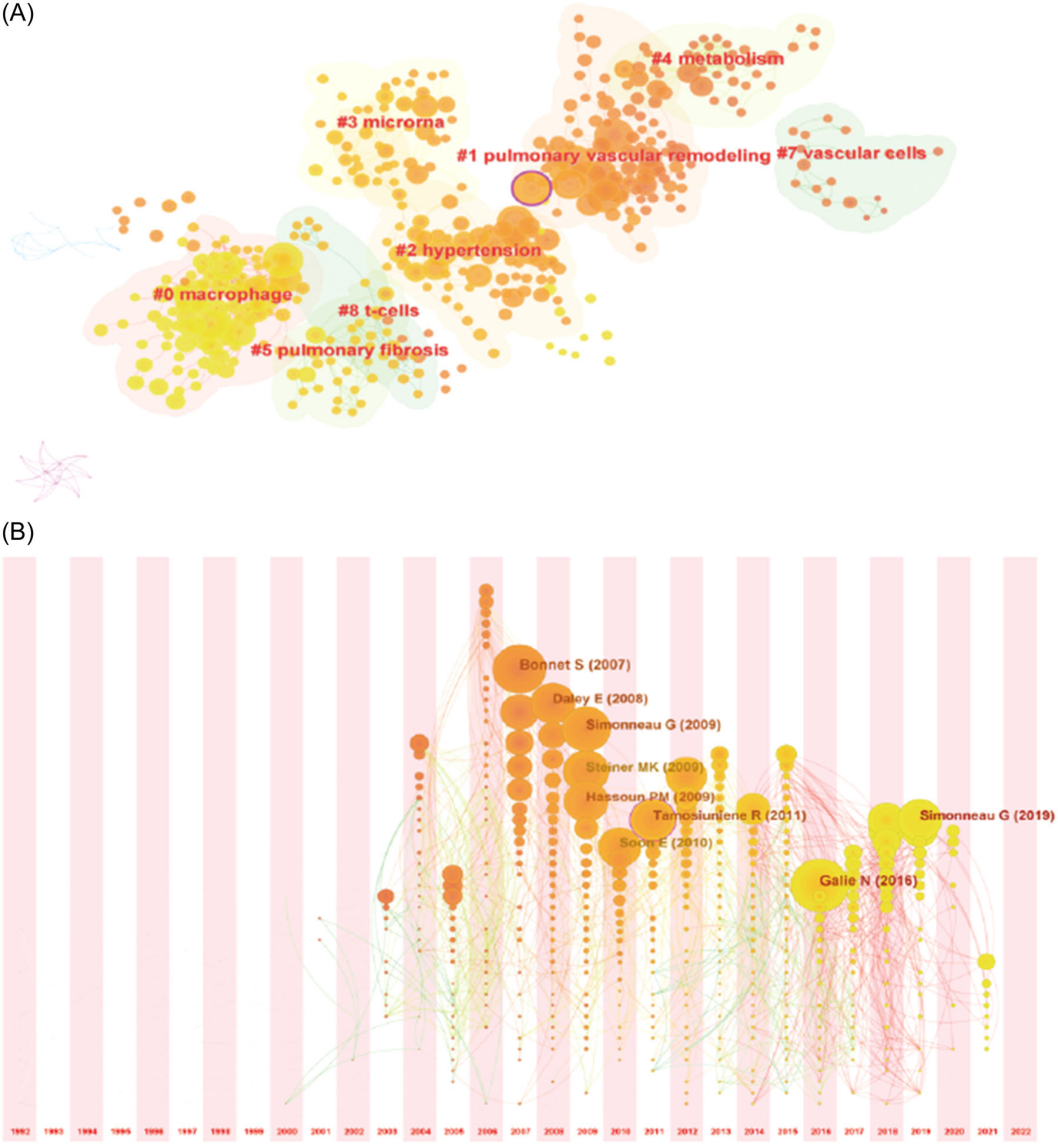
Analysis of reference co‐citation of T‐cell in pulmonary hypertension. (A) Cluster analysis of reference co‐citation of T‐cell in pulmonary hypertension. (B) The timezone view of reference co‐citation of T‐cell in pulmonary hypertension.

A cluster network of reference co‐citation with 787 codes and 1886 links was formed by CiteSpace 6.2.R3. The top 20 most cited references were summarized in Table 4. The most cited literature was published in the European Heart Journal by Galie N in 2016. The result of the reference co‐citation clustering analysis was displayed in Figure 6A. Arranged by cluster size, the top five cluster are “macrophage,” “pulmonary vascular remodeling,” “hypertension,” “microrna,” and “metabolism,” other clusters were “pulmonary fibrosis” and “vascular cells.” Timezone view of reference co‐citation was showed in Figure 6B, which reflected the changes in the research hotspots of the cited literature over time.

**Table 4:** The top 20 most cited references of pulmonary hypertension and T‐cell.

Ranking	Counts	Centrality	Author	Journal	Year	Vol	Page
1	39	0.03	Galie N	Eur. Heart. J.	2016	37	67
2	36	0.05	Bonnet S	P. Natl. Acad. Sci. USA	2007	104	11418
3	33	0.04	Simonneau G	J Am Coll Cardiol.	2013	62	34
4	31	0.02	Steiner MK	Circ. Res.	2009	104	236
5	30	0.1	Tamosiuniene R	Circ. Res.	2011	109	867
6	30	0.03	Daley E	J. Exp. Med.	2008	205	361
7	30	0.03	Hassoun PM	J. Am. Coll. Cardiol.	2009	54	10
8	30	0.01	Simonneau G	Eur. Respir. J.	2019	53	1801913
9	29	0.06	Soon E	Circulation	2010	122	921
10	28	0.03	Perros F	Am. J. Resp. Crit. Care.	2012	185	311
11	26	0.05	Taraseviciene‐stewart L	Am. J. Resp. Crit. Care.	2007	175	1280
12	25	0.01	Tamosiuniene R	Circ. Res.	2018	122	1689
13	24	0.02	Marsh LM	Eur. Respir. J.	2018	51	1701214
14	24	0.01	Humbert M	Eur. Respir. J.	2019	53	1801887
15	23	0.02	Rabinovitch M	Circ. Res.	2014	115	165
16	19	0.04	Perros F	Eur. Respir. J.	2007	29	462
17	19	0.03	Courboulin A	J. Exp. Med.	2011	208	535
18	19	0.01	Ulrich S	Respiration	2008	75	272
19	19	0	Thenappan T	BMJ‐Brit. Med. J.	2018	360	5492
20	17	0.01	Perros F	Eur. Respir. J.	2007	29	937

### 3.9 Burst detection of keywords and references

**Figure 7:**
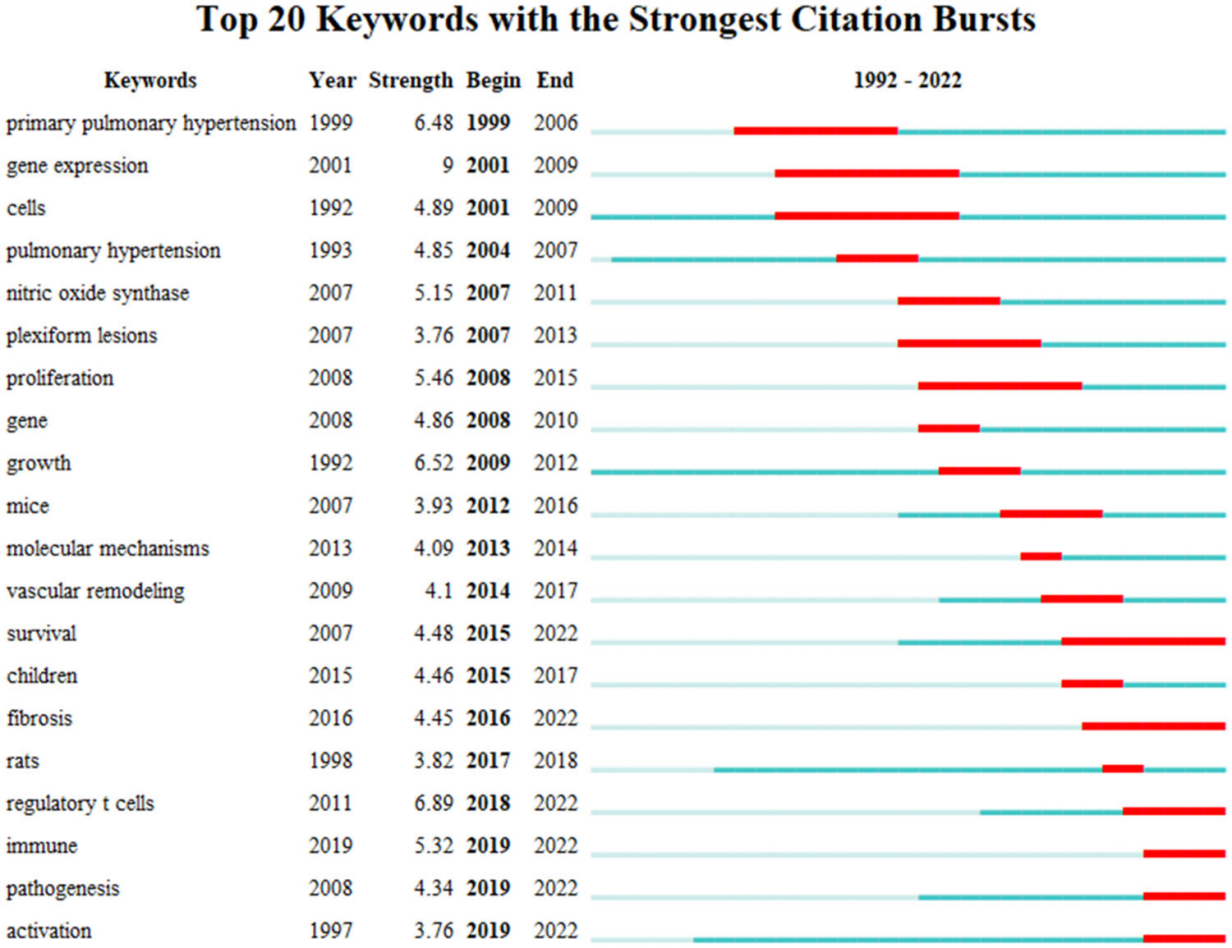
The burst detection of keywords of T‐cell in pulmonary hypertension.

**Figure 8:**
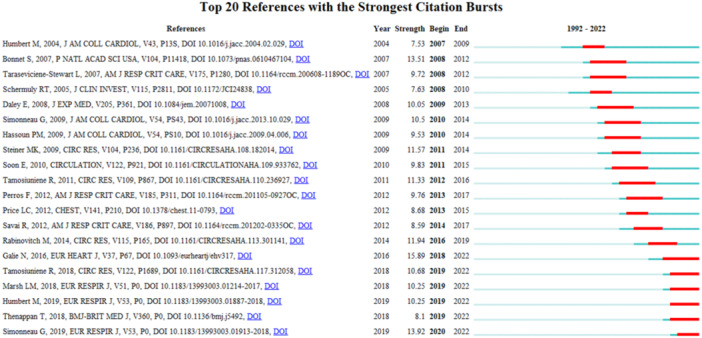
The burst detection of cited references of T‐cell in pulmonary hypertension.

The function of burst detection in CiteSpace can be used to detect the great changes of frequency of the keywords or cited literature in a short time. The burst detection of the top 20 keywords were shown in Figure 7, while the top 20 cited references were presented in Figure 8. They all listed by the burst time. The strongest citation burst and the longest duration burst of keyword are all “gene expression,” while t the most recently burst of keyword is “immune.” As for the burst of cited references, the strongest citation burst is “Galie N, 2016,” while the latest citation burst is “Simonneau G, 2019.”

## 4 DISCUSSION

A comprehensive understanding of a field could be obtained by using the bibliometric analysis method, which contributes a lot to subsequent research and provides clues for clinical treatment strategies. In this paper, we performed a bibliometric analysis of T‐cell in PH to explore its status of development. To the best of our knowledge, this study is the first bibliometric analysis to systematically analyze publications related to T‐cell in PH in the past 30 years.

PH is a complex pathophysiological disorder, which may involve multiple clinical conditions and may be associated with a variety of cardiovascular and respiratory diseases.^17^ Untreated, the prognosis for PH is poor, however, despite the growing numbers of available targeted medications, many PH patients continue to deteriorate and the disease ultimately remains fatal,^18^ owing to the unclear disease pathogenesis. Recent literatures have shown the important role of interaction between immune cells (both innate and adaptive immune cells) and stromal vascular cells in the process of pulmonary vascular remodeling.^19^ It has been demonstrated that T‐cells play a critical role in pulmonary vascular remodeling of PH,^20^ T‐cell deficiency could reverse the increased pulmonary arterial hypertension and vascular remodeling of PH.^21^ Among the helper T‐cell subtypes, we have proved the role of Th17 response in the development of HPH, targeting Th17 cytokine interleukin‐17 (IL‐17) reversed hypoxia‐induced pulmonary vascular remodeling.^22^ Other researchers also showed the central role of Th17 cells in hypoxia‐induced PH.^13^, ^23^ Th2 immune response can also induce severe pulmonary arterial remodeling, Th2 immune response regulated signals for cell proliferation, differentiation of smooth muscle actin‐positive cells, and rearrangement of the cellular organization resulting in a severely remodeled arterial wall, and inhibiting Th2 response and Th2 cytokine interleukin 13 significantly ameliorated pulmonary arterial muscularization.^24^ In contrast, Th1 immune responses, which secrete cytokines such as interferon‐gamma and tumor necrosis factor‐α, appear to have little connection with the development of pulmonary vascular remodeling in PH, Wang et al. reported a decrease in the percentage of monocyte‐derived dendritic cells and higher concentrations of serum interleukin‐12 in the peripheral blood of IPAH patients, suggesting a Th1 immune reaction in the pathogenesis of IPAH.^25^ For regulatory T‐cells (Tregs), Tregs can regulate PH initiation and progression by secreting positive cytokines, interacting directly or indirectly with other immune cells to ameliorate PAEC injury, regulate PASMC proliferation and apoptosis, control fibroblast proliferation and activation, and maintain immune homeostasis, then play a protective role in multiple pathological processes of PH.^12^, ^26^, ^27^, ^28^ Disproportionate Treg/Th17 ratios (reduced Tregs and increased Th17 cells) have been reported in various PH, which has been identified as positively correlated with PH severity and prognosis.^29^, ^30^ Except CD4 + T‐cells, CD8+ cytotoxic T‐cells also participated in PH, CD8 + T‐cell depletion could deteriorate disease process of PH.^31^ It was reported that circulating cytotoxic CD8 + T‐cells were decreased in IPAH patients,^32^ and CD8 + T‐cells subtypes also altered.^11^ CD8 + T‐cell infiltrated in perivascular pulmonary vasculature is associated with impaired endothelial function in PH.^33^ Hence, T‐cell‐immunity is a crucial mechanism of PH pathogenesis, and targeting T‐cells is of decisive significance for the prognosis of PH,^34^ which needs our attention and concern. Then this present study brings us new ideas for the T cell‐immunity related studies in PH and may benefit further research works on the immunological etiology, diagnosis, and T cell‐targeting treatment of the disease.

Over the past few decades, the quantity of T‐cells in PH publications shows an overall upward trend, demonstrating that T‐cell immunity has attracted more and more attention as an important mechanism in the pathogenesis of PH. In terms of the authors, Voelkel Norbert F. and Humbert Marc were the leaders in the publication; and professors such as Perros Frédéric also have made outstanding contributions in this field.

Our analyses revealed that the majority of studies in this field published during this period were from the United States (43.1%), followed by China (17.4%), France (8.9%), Germany (8.5%), England (6.4%), and Japan (5.9%). The number of papers published in a certain research field is considered to be a significant index to appraise the scientific research level of a country or institution, our results indicated the powerful scientific research ability of the United States and Europe, Asia countries like China and Japan are also powerhouse of scientific research in this field. Because the relative high incidence and prevalence of PAH in France,^35^ organizations from France predominate in this field, including Udice French Research University, Institut National de la Santé et de la Recherche Médicale and Assistance Publique—Hôpitaux de Paris, which are all the prestigious research institutions in the world that are committed to scientific research in this field. Our results provide a reference for scholars to choose an appropriate country or institution to communicate, cooperate and learning in this field.

The top three journals that published the largest proportion of the studies in this analysis included the *American Journal of Physiology Lung Cellular and Molecular Physiology* (44), *American Journal of Respiratory and Critical Care Medical* (27), *American Journal of Respiratory Cell and Molecular Biology* (20). Basic scientific research was the cornerstone and motivity to promote the development of a specific field, and these journals mainly focus on basic scientific research in respiratory research field which lead the progress of T‐cell immunity in PH research in this era. These journals are thus likely to continue publishing important discoveries in this field in the near future and give researchers a dependable reference to search the literature and submit manuscripts.

Keywords can reflect immediate information about the topic in certain research.^36^ The clusters represent the keywords or terms that appear most frequently in the literature, therefore reflect the hotspots in a period.

In the TOP 20 keywords, there are three keywords about t cell: “T‐cells,” “Regulatory t cells,” and “Activated t cell,” indicating the important role of T‐cell immunity in the pulmonary vascular remodeling development of PH which we mentioned previously. Recent reports demonstrated that increase in T‐cell activation induced by immune checkpoint inhibitor therapy can cause PH,^37^, ^38^ so physicians should be aware of pharmacovigilance of these drugs. The mechanisms of T‐cell immunity activation and T‐cell immunity mediated pulmonary vascular remodeling remains unclear, which need further research. In the keywords co‐citation clustering analysis, cluster 0 “chronic hypoxia” told us the research about T‐cell immunity in PH mainly focused on Group 3 PH, and relevant research works are needed in other types of PH, especially Group 1 PAH. It is known that acute hypoxia causes pulmonary arterial contraction and elevated pulmonary artery pressure, while chronic hypoxia results in pulmonary vascular remodeling, medial thickening, and extension of smooth muscle into partially muscular arteries, hypoxia‐inducible factor (HIF) pathway is the major cellular oxygen‐sensing mechanism implicated during this process.^39^ Cluster 8 “nitric oxide” in cluster analysis of keyword co‐appearance and cluster 1 “pulmonary vascular remodeling” in cluster analysis of reference co‐citation reveal that vascular tone play an important role in PH,^40^ which is an important treatment target for PH.

Cluster 3 “microRNA” of reference co‐citation clustering analysis in Figure 6A demonstrated the possible T‐cell immunity activation mechanisms in PH. As new therapeutic targets for PH, “miRNAs” can participate in the pathophysiology of PH in many ways.^41^ miR‐15 expression was reported to increase the induction of T‐cells.^42^ miR‐483 might reduce experimental PH by inhibition of multiple adverse inflammatory responses.^43^ More research works are needed to confirm the regulatory effect of miRNAs on T‐cells in PH and the potential mechanisms.

According to the burst detection displayed in Figure 7, keywords related to T‐cell immunity mechanisms in PH have been frequently mentioned. Terms such as “vascular remodeling,” “growth,” and “fibrosis” showed that T‐cells interact with stromal vascular cells to induce pulmonary vascular remodeling. Endothelial cell dysfunction is an initial stage of pulmonary vascular remodeling, and there is complex crosstalk between endothelial cells and T‐cells in vascular disease.^44^ Cytotoxic T‐cells could induce the apoptotic death of endothelial in systemic sclerosis, which may be the mechanism of CTD‐PAH.^45^ While Tregs provide cytoprotection for endothelial cells through secretion of IL‐10,^46^ Tregs could prevent PH via improving endothelial injury.^27^ Conversely, endothelial cells also can regulate the T‐cell function, endothelial cells play an active role in the immune cell activation, including supporting T‐cell proliferation and increasing Treg suppressor function.^47^ Pulmonary vascular endothelial cell injury could also lead an increased perivascular CD4 + T‐cell infiltration and then participates in pulmonary vascular remodeling.^48^ For smooth muscle cells (SMCs), there is also cross‐talk with T‐cells, CD4 + T‐cells can reorganize vascular SMCs cell membrane to assemble an immunologic synapse with SMCs,^49^ and affect SMCs phenotype and proliferation.^50^ T‐cell immunity response could lead to chemokines production by smooth muscle cells which in turn inhibits endothelial healing.^51^ SMCs in turn affect CD4 + T cell proliferation too.^52^ The adventitial layer is physiologically complex and consists of numerous cell types including fibroblasts and immune cells, which is an inflammatory signaling hub. Interaction between T‐cells and fibroblasts was recognized as early as 1980s.^53^ T cell‐derived IL‐17A promotes proliferation of fibroblasts and subsequent vascular fibrosis.^54^ Conversely, fibroblasts play important roles in modulating T cell recruitment, differentiation and function.^55^ Therefore, a more precise understanding of the interrelation between stromal vascular cells and T‐cells will enable a better future therapeutic design by targeting this interrelationship, so the mechanisms of this interaction need to be clarified further.

Burst detection of references provides most influential articles for researcher in this field. As shown in Figure 8, the article published by Galie N in 2016 has the highest burst strength 15.89. It shows that this author took the lead in the quality of the publications, making great contributions to the research in the field of T‐cell immunity in PH. The works of this author would benefit many researchers in this related field.

This study systematically summarized the current achievements and prospects in the field of T‐cell immunity in PH, but there are also several limitations in this study, which must be taken into account when clarifying the results. First, we only include English written literature, it is hard to prevent bias in the results. The second limitation is incomplete literature collection, the literatures were only collected from the WoSCC, data from other databases have not been included. Third, incomplete extraction of a few isolated keywords by software may affect the accuracy of the results.

## 5 CONCLUSION

This work provides readers with a scientific and comprehensive view of the structure of knowledge in the field of T‐cell immunity in PH, providing research hotspots, frontiers, development trends and a theoretical basis for subsequent scientific exploration. In particular, our visual results give readers an accessible and intuitive impression of the status of T‐cell immunity ‐related research in PH, which will benefit many researchers.

## AUTHOR CONTRIBUTIONS

Xian Chen contributed to conception and design of the study. Lei Wang and Xian Chen collected data from the database. Lei Wang wrote the manuscript. All authors contributed to manuscript revision, read, and approved the submitted version.

## Data Availability

All data generated or analyzed during this study are included in this published article, and are available from the corresponding author on reasonable request.
